# Supplementary figures and images for: A covariation analysis reveals elements of selectivity in quorum sensing systems
Source: eLife. 2021 Jun 28;10:e69169. doi: 10.7554/eLife.69169 (PMC8328516; doi:10.7554/eLife.69169)

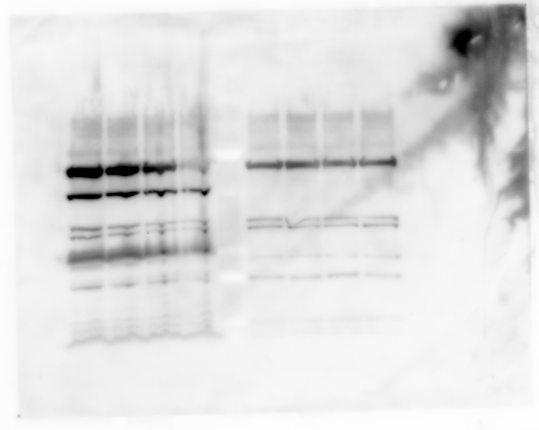

Supplement: Figure 4—figure supplement 2—source data 1. — Lanes 1–4 are not relevant and not shown in any figure. Lane 5 contains a protein ladder. Lanes 6–9 contain the soluble fraction of lysed cultures of PAO-SC4 with the following LasR amino acid substitutions: (6) wild type (WT), (7) A127L, (8) L130F, (9) R61L. [file elife-69169-fig4-figsupp2-data1.png.zip › Figure4-figuresupplement2–sourcedata1.png]

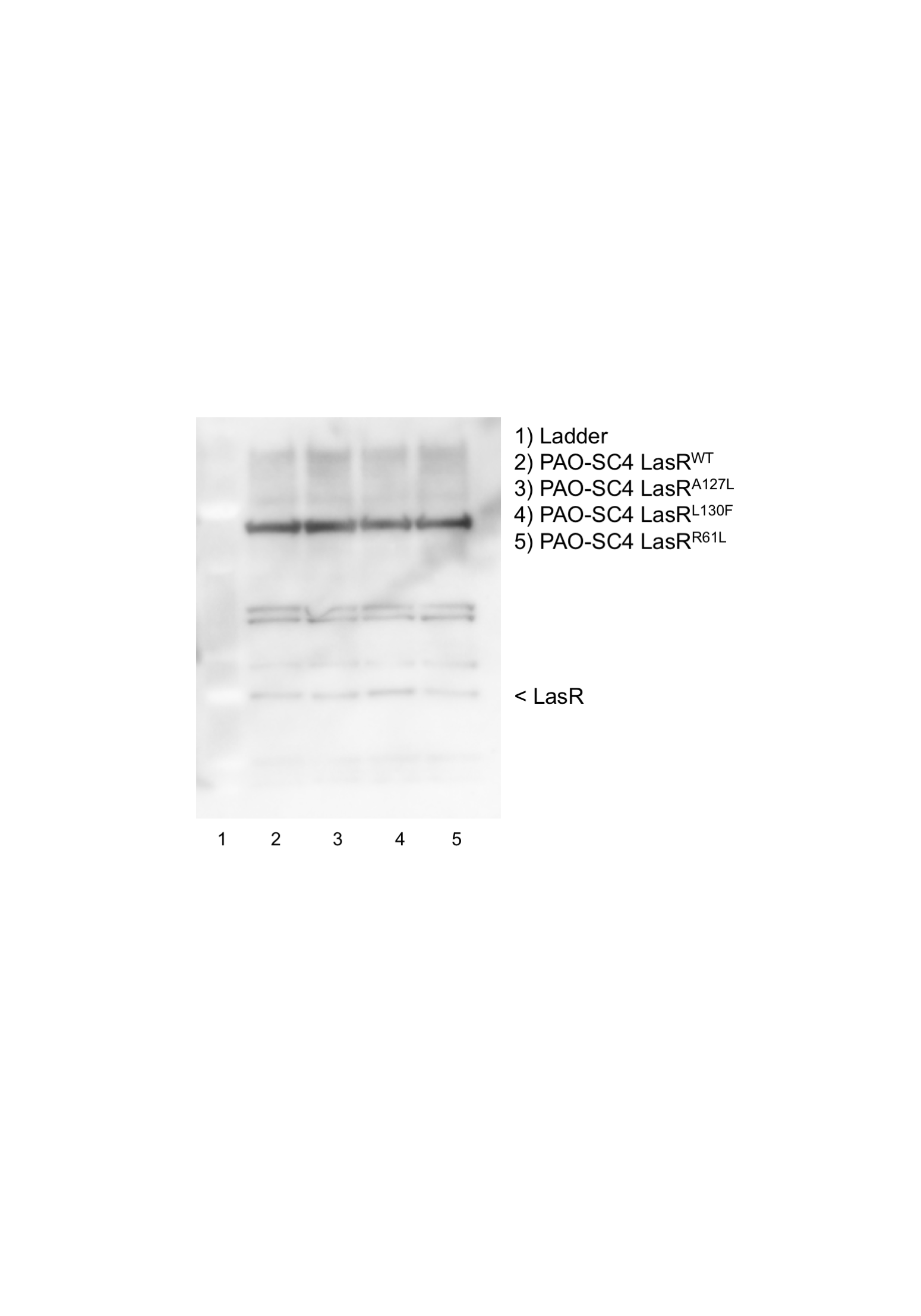

Supplement: Figure 4—figure supplement 2—source data 2. [file elife-69169-fig4-figsupp2-data2.tiff.zip › Figure4-figuresupplement2–sourcedata2.tiff]

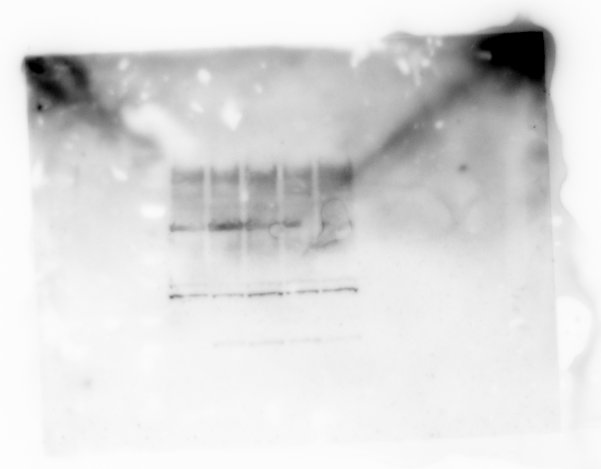

Supplement: Figure 4—figure supplement 2—source data 3. — Lane 1 contains a protein ladder. Lane 2 contains the soluble fraction of lysed Pseudomonas aeruginosa PAO1∆lasR. Lanes 3–6 contain the soluble fraction of lysed cultures of PAO-SC4 with the following LasR amino acid substitutions: (3) wild type (WT), (4) A127L, (5) L130F, (6) R61L. [file elife-69169-fig4-figsupp2-data3.png.zip › Figure4–figuresupplement2–sourcedata3.png]

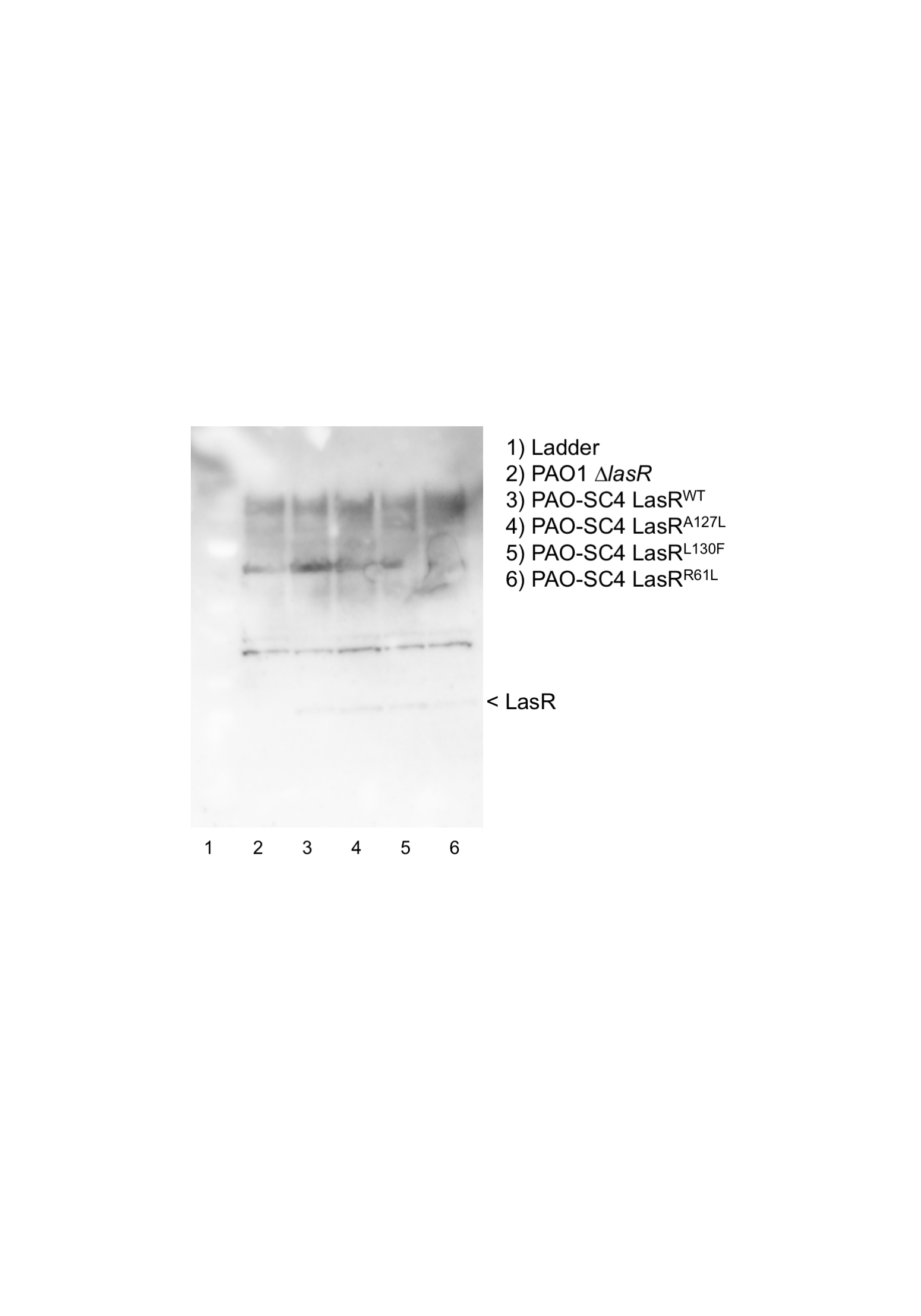

Supplement: Figure 4—figure supplement 2—source data 4. [file elife-69169-fig4-figsupp2-data4.tiff.zip › Figure4–figuresupplement2–sourcedata4.tiff]

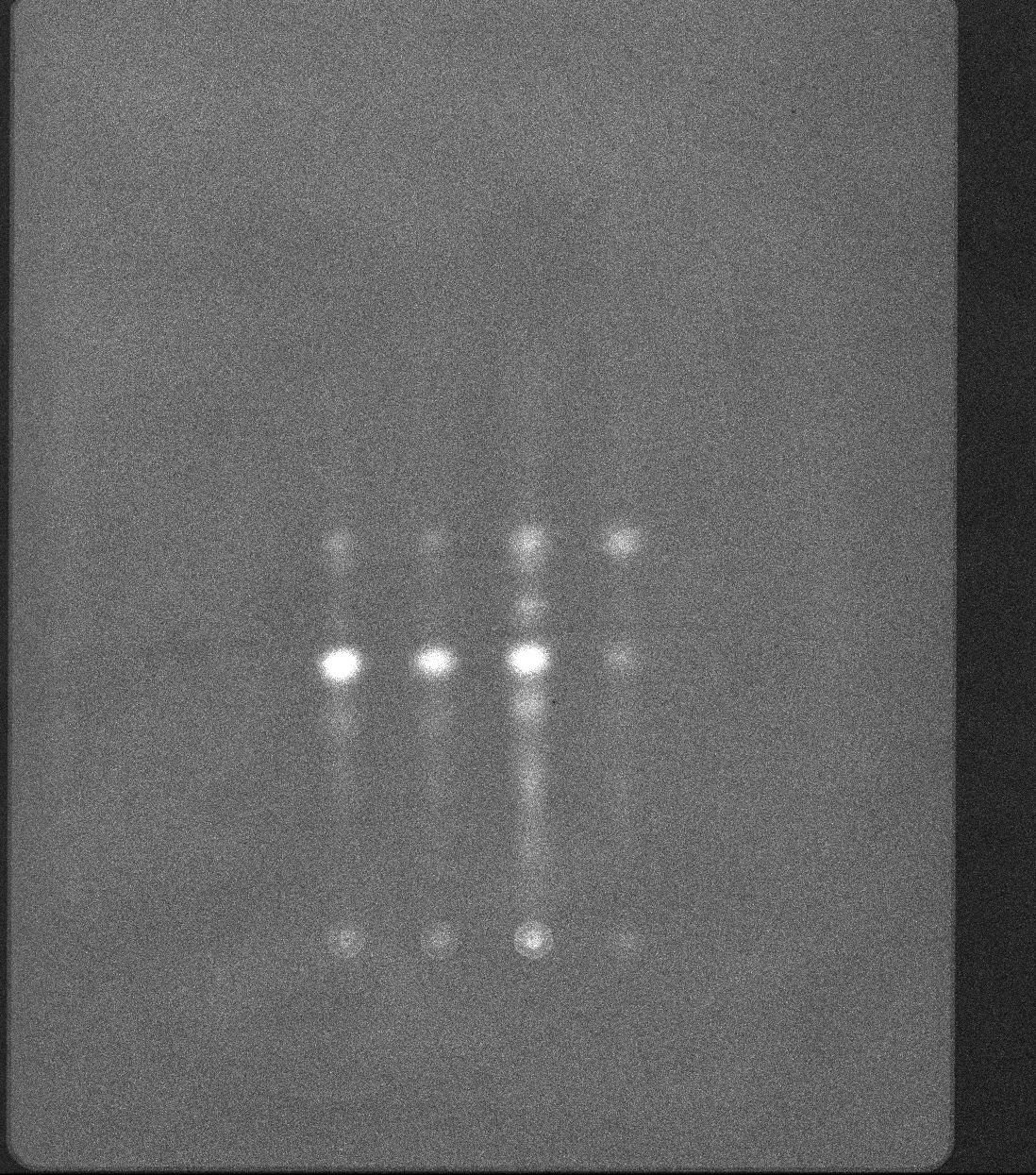

Supplement: Figure 5—figure supplement 1—source data 1. — This TLC contained six samples. 14C-AHLs were extracted from (1) media-only control, (2) Pseudomonas aeruginosa PAO1ΔrhlI, (3) PAO-SC4 pJN-lasIWT, (4) PAO-SC4 pJN-lasI140Y, 145S, 152L, 157V, (5) PAO-SC4 pJN-lasI125I, 140Y, (6) PAO-SC4 pJN-empty. Lanes 1 and 6 have no detectable radioactivity, lanes 4 and 5 are irrelevant and not shown in any figure. [file elife-69169-fig5-figsupp1-data1.jpeg.zip › Figure 5 – figure supplement 1- source data 1.jpeg]

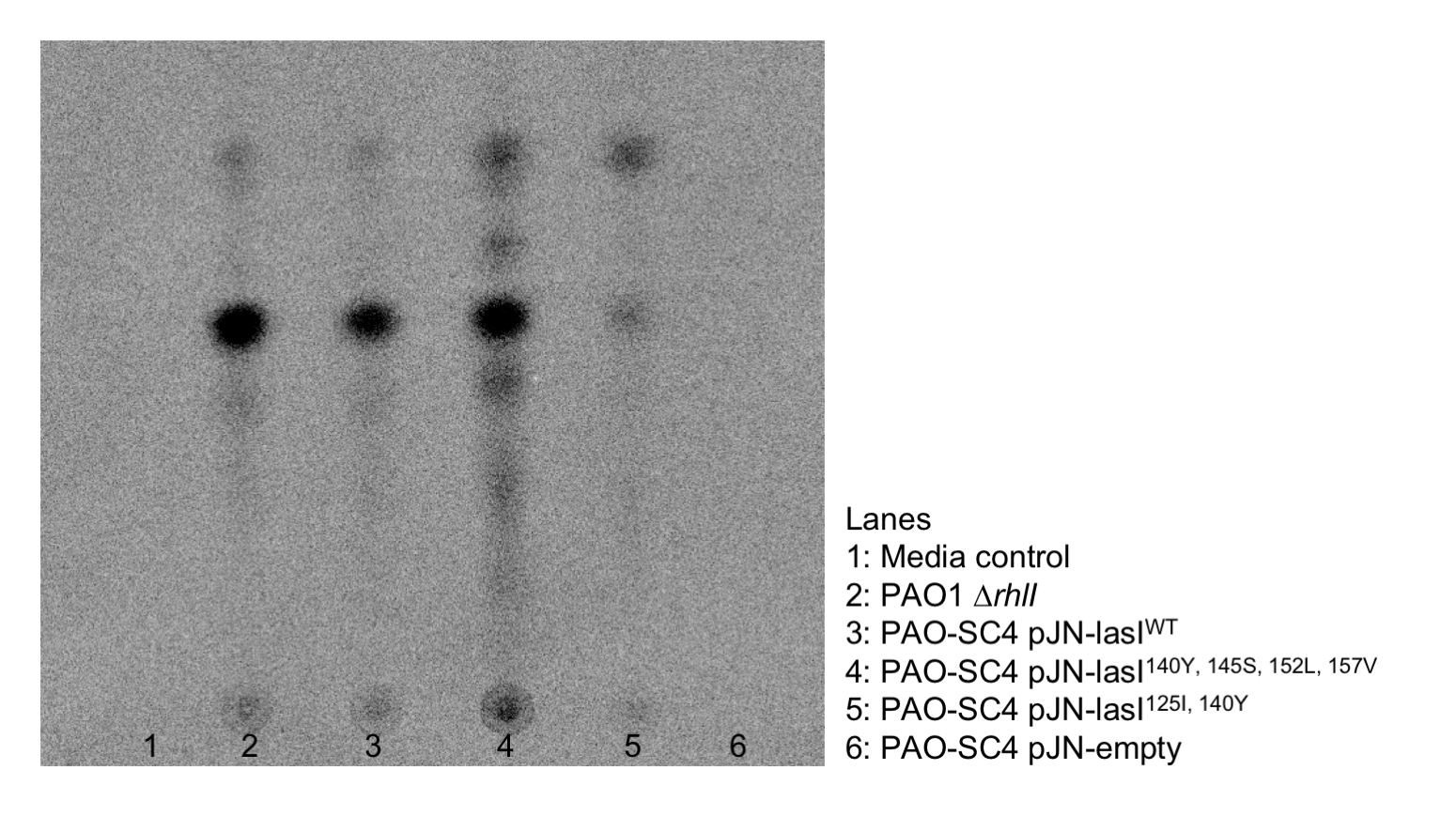

Supplement: Figure 5—figure supplement 1—source data 2. [file elife-69169-fig5-figsupp1-data2.tiff.zip › Figure5–figuresupplement1-sourcedata2.tiff]

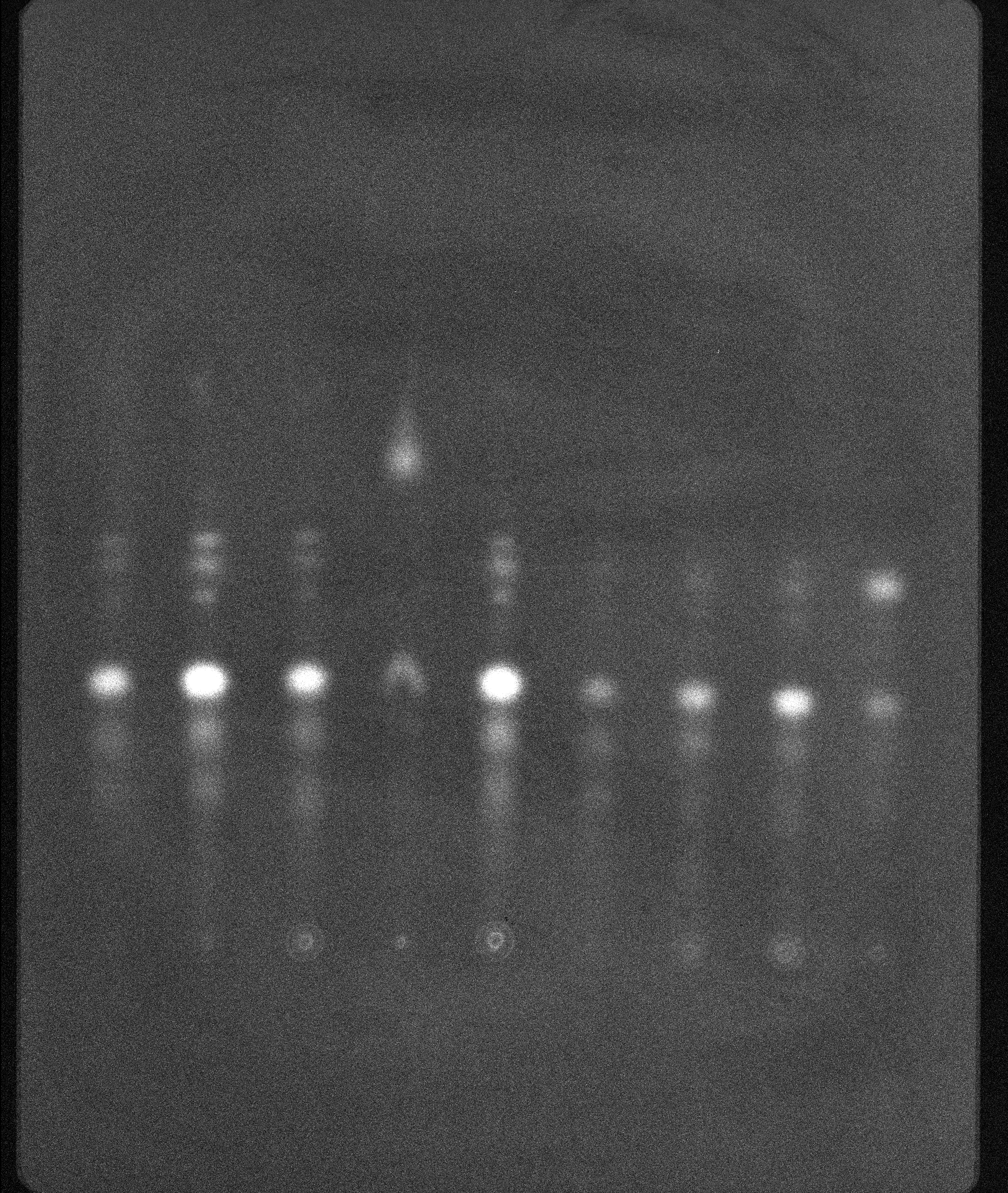

Supplement: Figure 5—figure supplement 1—source data 3. — This TLC contained nine samples. 14C-AHLs were extracted from PAO-SC4 harboring pJN-lasI with the following amino acid substitutions: (1) wild type (WT), (2) 145S, (3) 152L, (4) 157V, (5) 145S, 152L. Lanes 6–9 are not relevant and not shown in any figure. Lanes from this TLC are also shown in Figure 7—figure supplement 2E as the second set of images in a series of three TLC images. [file elife-69169-fig5-figsupp1-data3.jpg.zip › Figure5–figuresupplement1-sourcedata3.jpg]

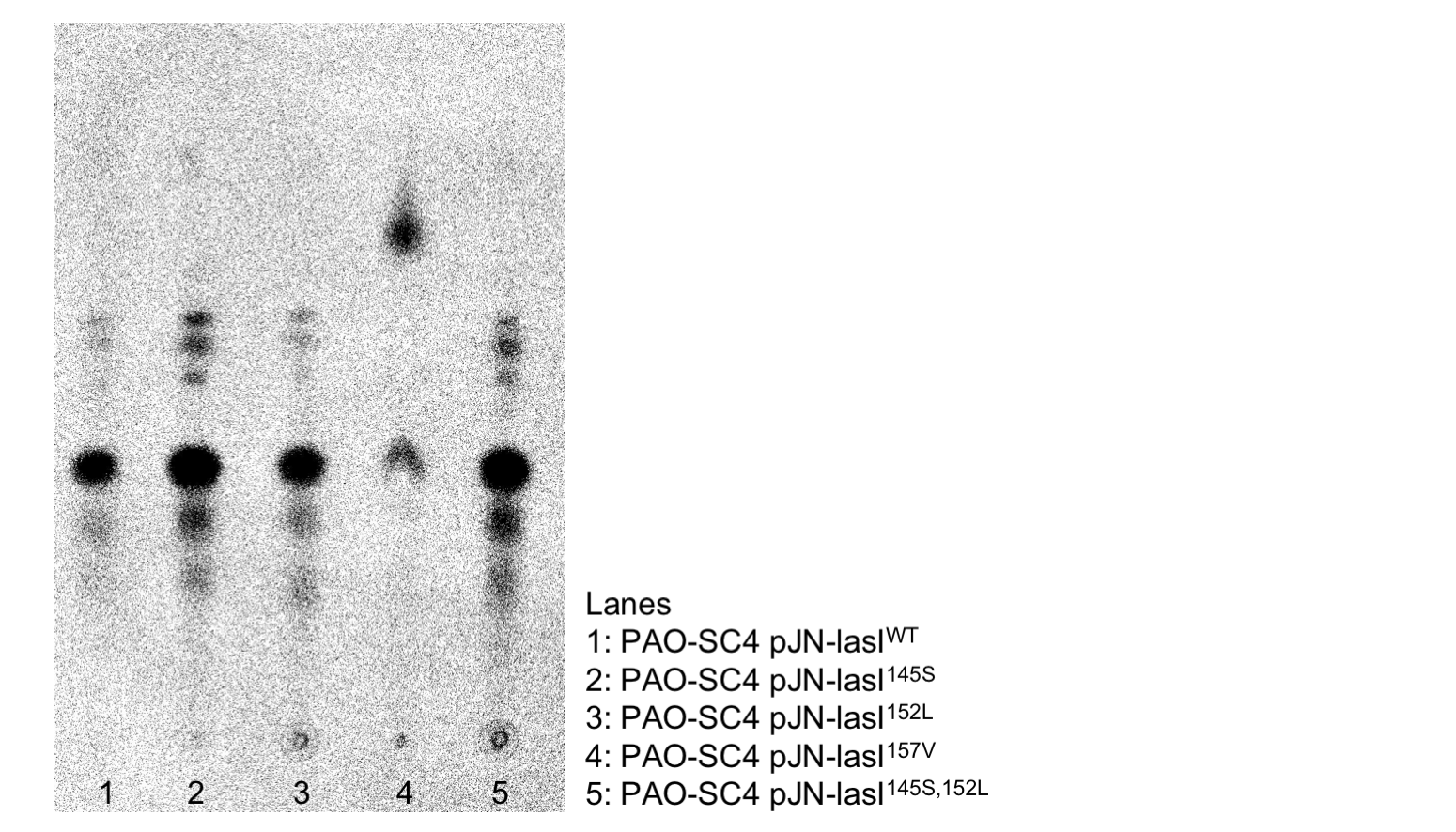

Supplement: Figure 5—figure supplement 1—source data 4. [file elife-69169-fig5-figsupp1-data4.tiff.zip › Figure5–figuresupplement1-sourcedata4.tiff]

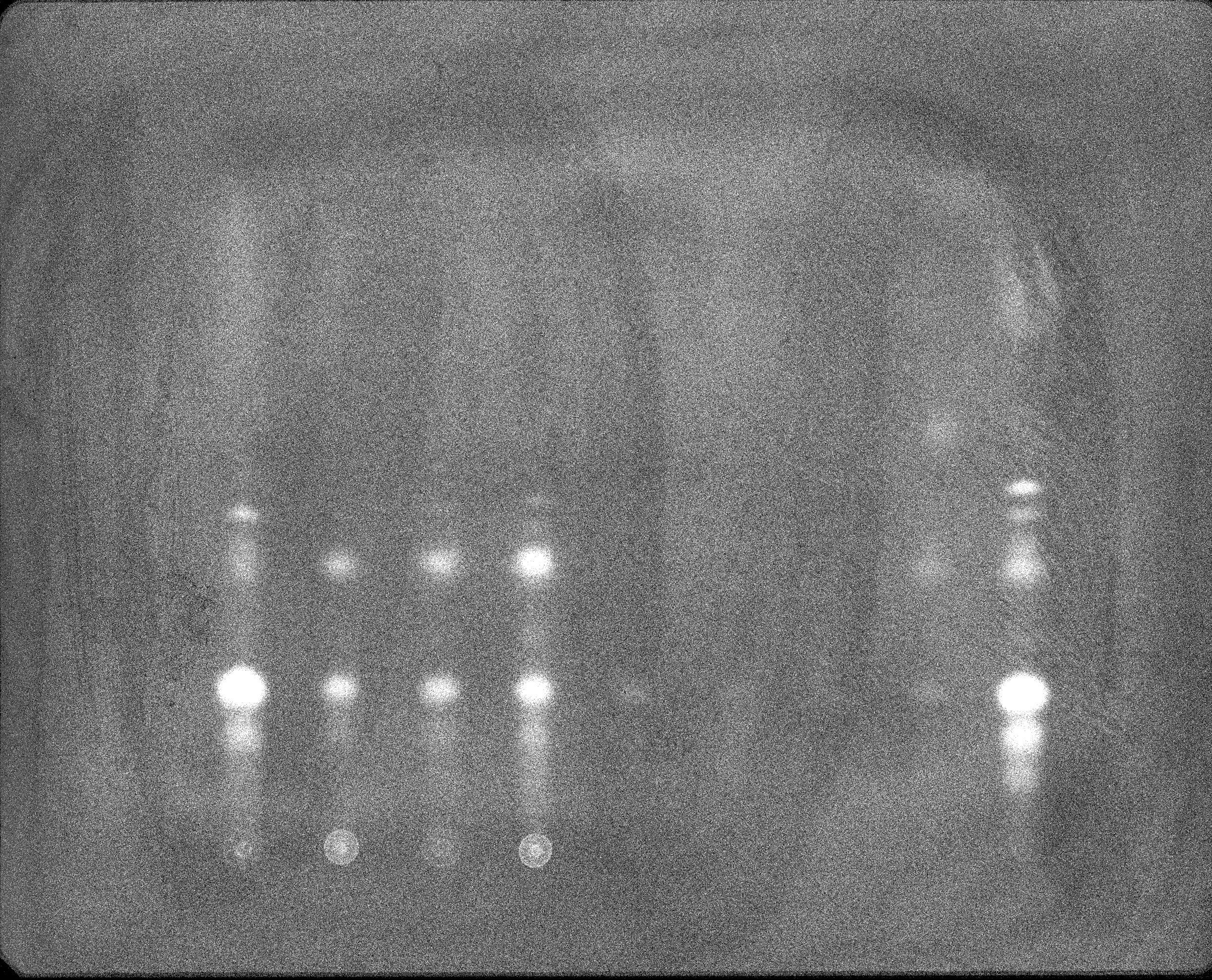

Supplement: Figure 5—figure supplement 1—source data 5. — This TLC contained nine samples. 14C-AHLs were extracted from PAO-SC4 harboring pJN-lasI with the following amino acid substitutions: (1) wild type (WT), (2) 125I, (3) 125I, 152L, (4) 125I, 145S, 152L, (5) 102M, (6) 142G, (7) 145P, (8) 157W, (9) 140Y. Lanes 1–4 of this TLC are also shown in Figure 7—figure supplement 2E as the first image in a series of three TLC images. Lane 9 is irrelevant and not shown in any figure. [file elife-69169-fig5-figsupp1-data5.jpeg.zip › Figure5–figuresupplement1-sourcedata5.jpeg]

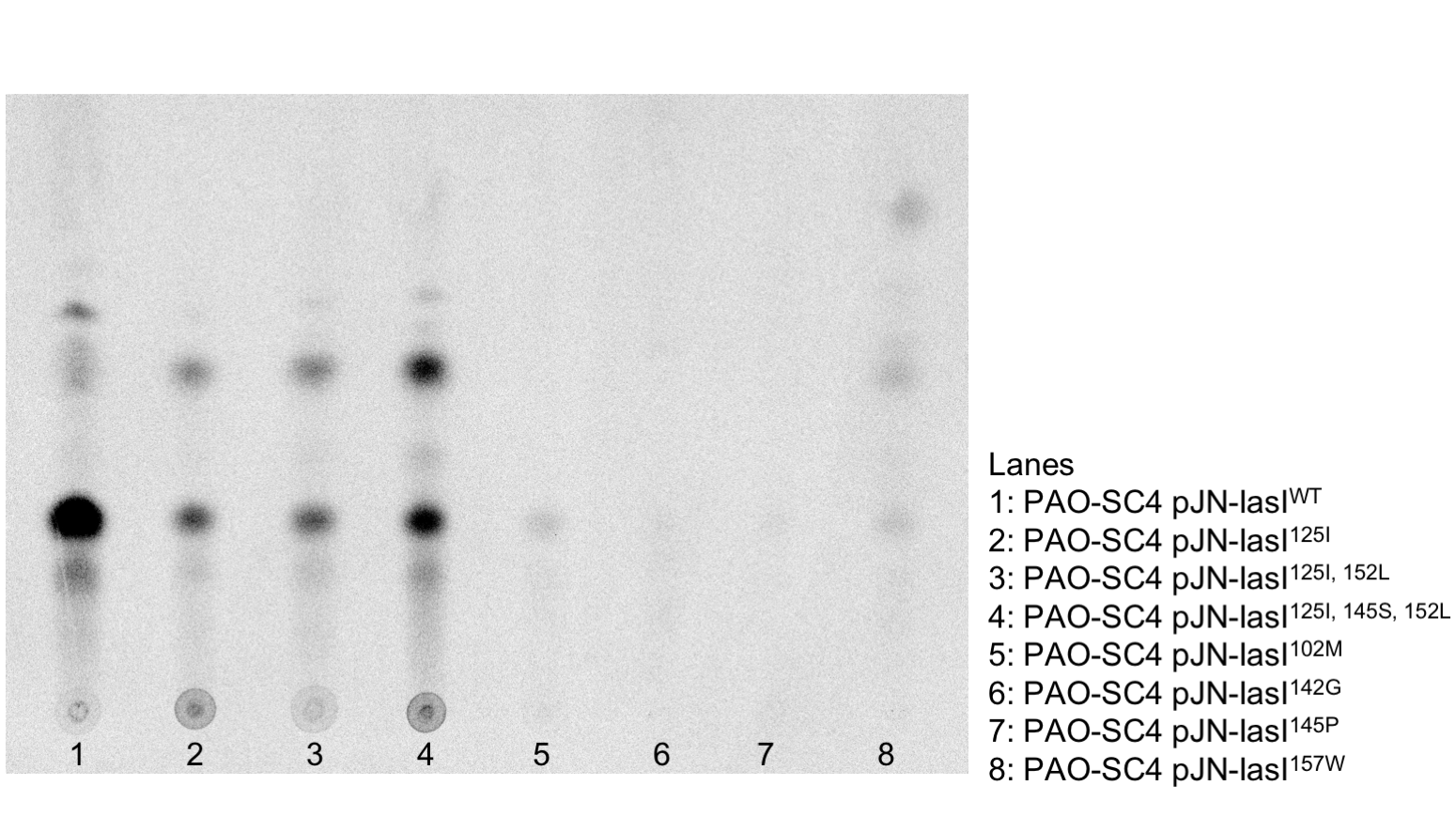

Supplement: Figure 5—figure supplement 1—source data 6. [file elife-69169-fig5-figsupp1-data6.tiff.zip › Figure5–figuresupplement1-sourcedata6.tiff]

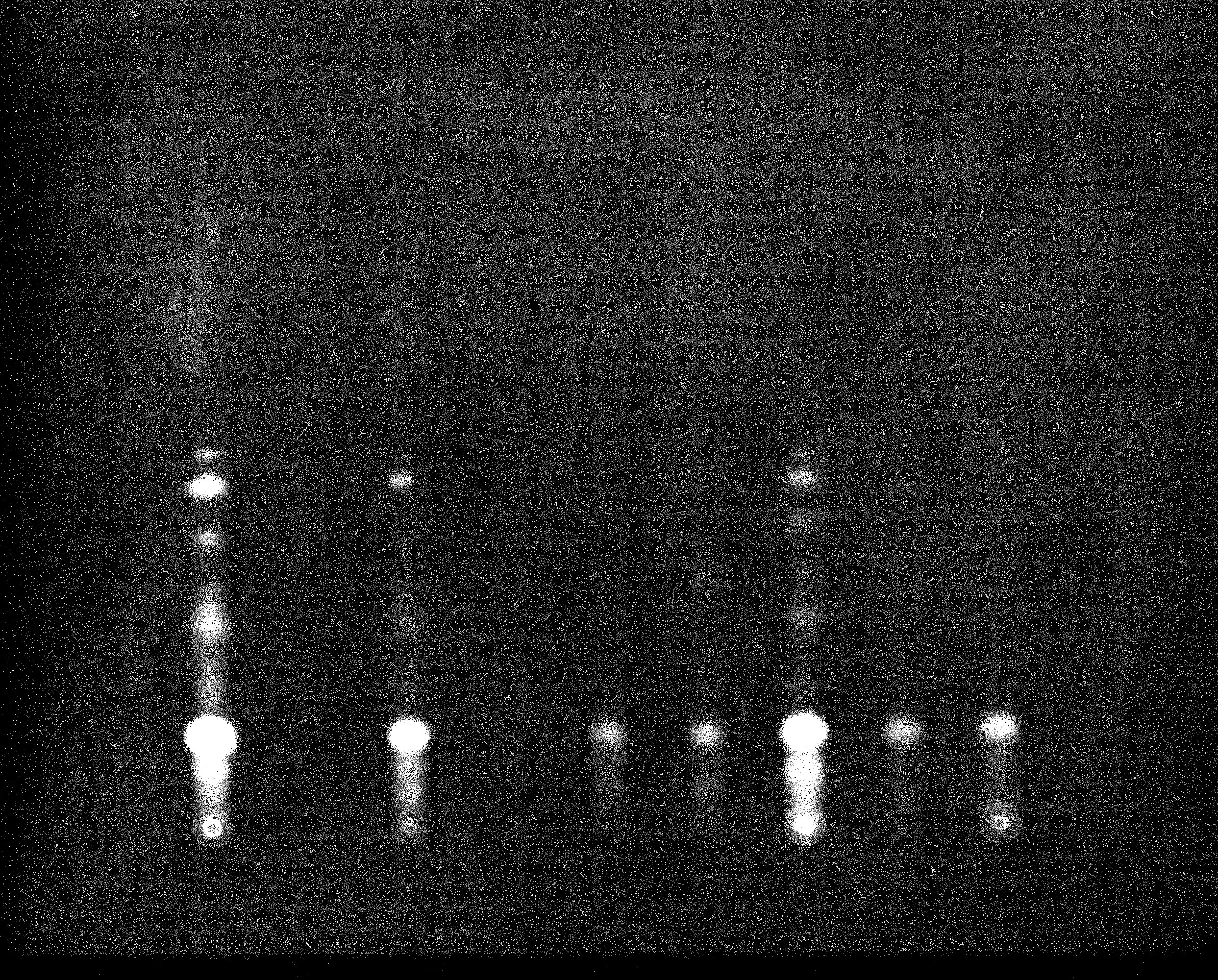

Supplement: Figure 5—figure supplement 1—source data 7. — This TLC contained nine samples. 14C-AHLs were extracted from: (1) Pseudomonas aeruginosa PAO1ΔrhlI, (2) PAO-SC4 pJN-empty, (3) PAO-SC4 pJN-lasIWT, (4) PAO-SC4 pJN-lasI102S, (5) PAO-SC4 pJN-lasI102I, (6) PAO-SC4 pJN-lasI142A, (7) PAO-SC4 pJN-lasI145A, (8) PAO-SC4 pJN-lasI145D, (9) PAO-SC4 pJN-lasI157V. Lanes 3–9 are shown with labels in Figure 5—figure supplement 1E. Lanes 1 and 2 are not shown in any figure. [file elife-69169-fig5-figsupp1-data7.jpeg.zip › Figure5–figuresupplement1-sourcedata7.jpeg]

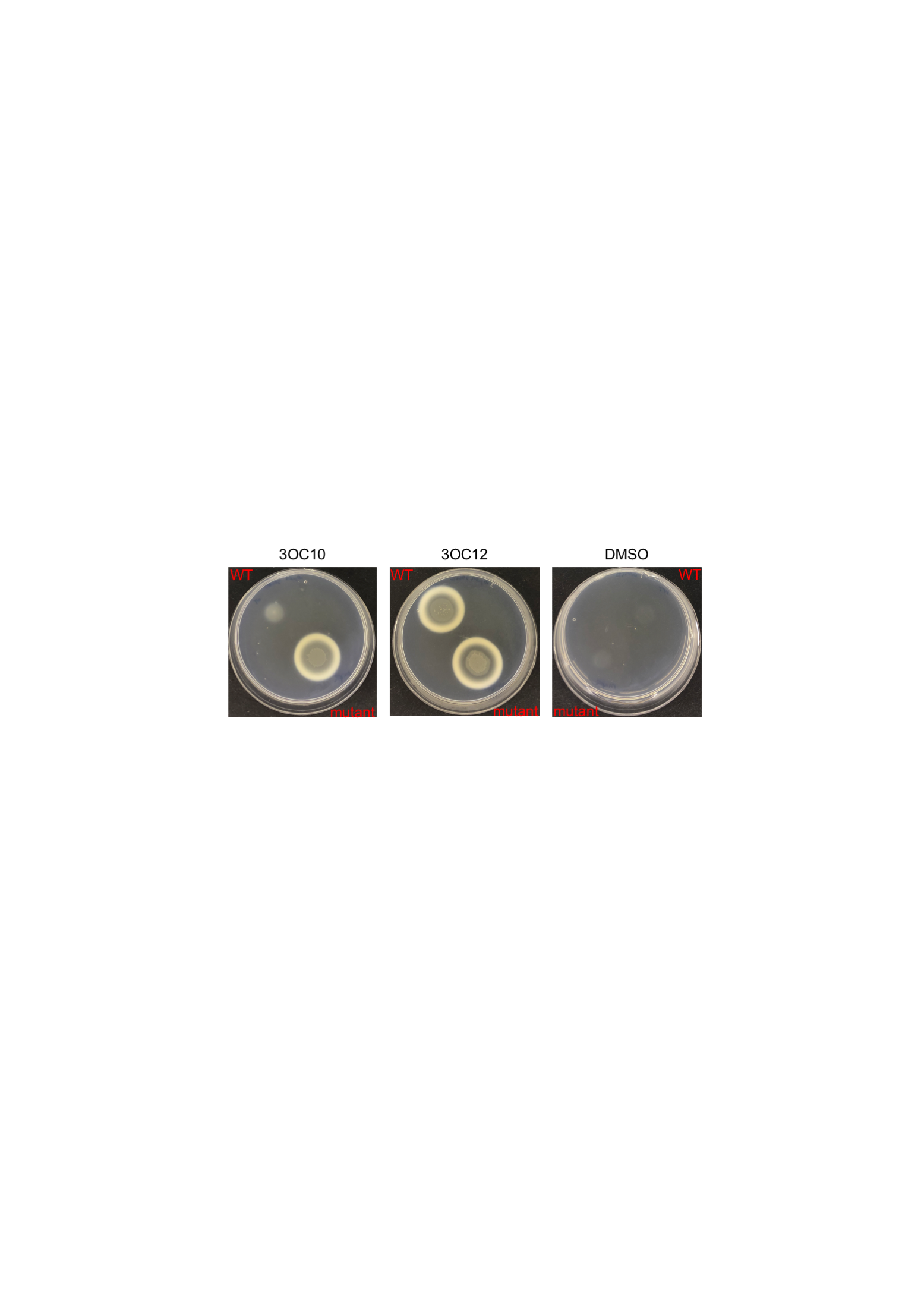

Supplement: Figure 6—source data 1. — Pseudomonas aeruginosa PAO-SC4 wild type (WT) or harboring LasRL125F, A127M, L130F are labeled as ‘WT’ or ‘mutant’, respectively. Plates are 60 mm × 15 mm. [file elife-69169-fig6-data1.tiff.zip › Figure6sourcedata.tiff]

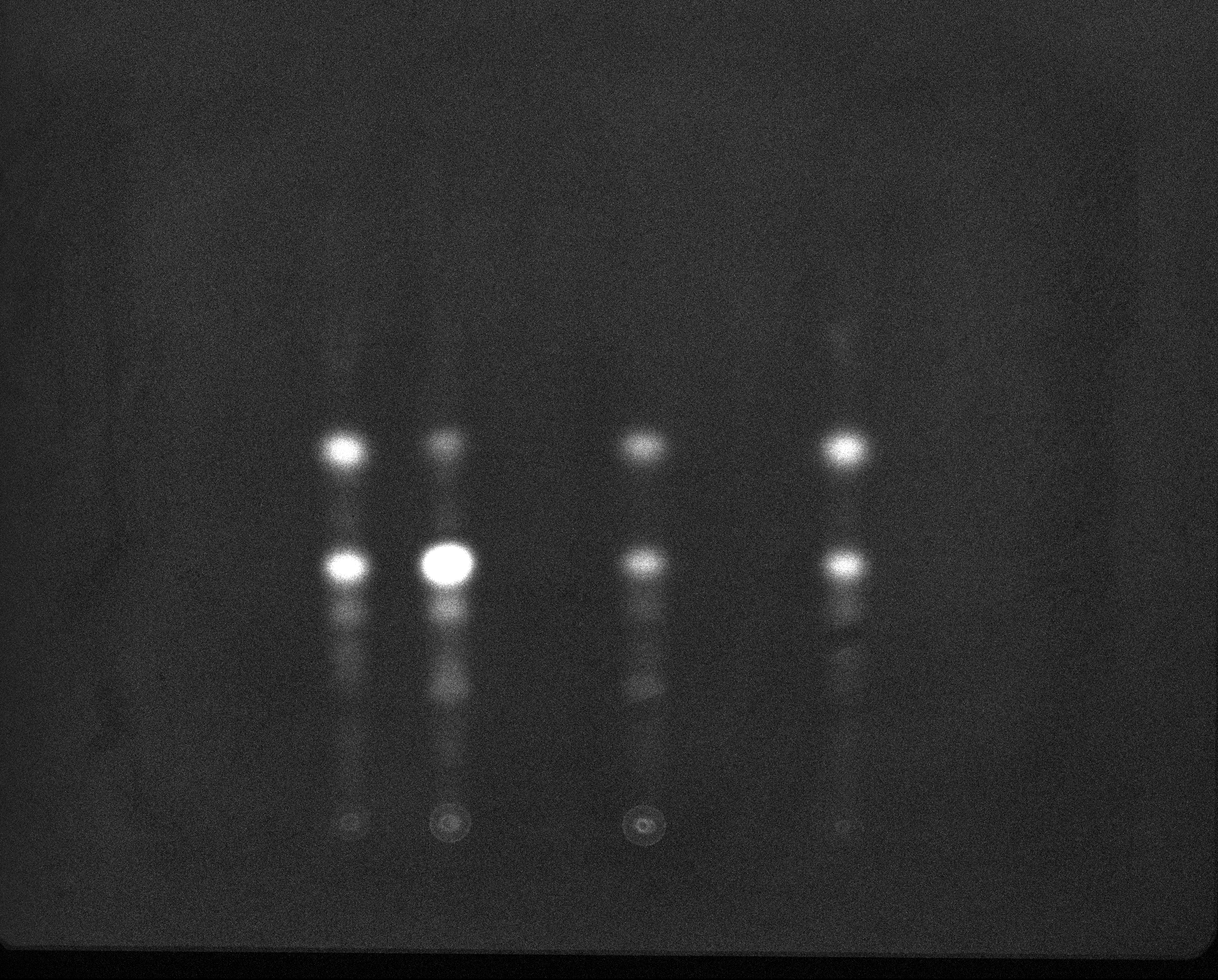

Supplement: Figure 7—figure supplement 2—source data 1. — Source data for the first two sets of images in this series are provided in Figure 5—figure supplement 1—source data 3–6. Source data for the third image is provided here. This TLC contained seven samples. 14C-AHLs were extracted from PAO-SC4 harboring pJN-lasI with the following amino acid substitutions: (1) 125I, 145S, (2) wild type (WT). These two lanes are shown, with labels in Figure 7—figure supplement 2E. Lanes 3–6 are irrelevant and not shown in any figure. [file elife-69169-fig7-figsupp2-data1.jpg.zip › Figure7-figuresupplement2-sourcedata.jpg]
