# Supplementary material for: A covariation analysis reveals elements of selectivity in quorum sensing systems
Source: eLife. 2021 Jun 28;10:e69169. doi: 10.7554/eLife.69169 (PMC8328516; doi:10.7554/eLife.69169)
Supplement: Supplementary file 1. — (A) Manually curated quorum sensing (QS) synthases and receptors. (B) Select previously reported data for LasR homologs with relevant amino acid substitutions. The native signal for each receptor is indicated in parentheses. (C) Select previously reported data for LasI homologs with relevant amino acid substitutions. The native signal for each synthase is indicated in parentheses. (D) LasR amino acid substitutions evaluated in Figure 3. Relative abundance is the frequency of a given amino acid at the indicated position across all LasR homologs. (E) LasI amino acid substitutions evaluated in Figure 5—figure supplement 1. Relative abundance is the frequency of a given amino acid at the indicated position across all LasI homologs. (F) Primers used in this study. [file elife-69169-supp1.docx]

**Supplementary File 1A | Manually curated QS synthases and receptors.**

| QS system name | Bacterium | Cognate signal | GenBank (I/R) |
| --- | --- | --- | --- |
| LasI/R^a^ | *Pseudomonas aeruginosa* PAO1 | 3OC12-HSL | AAG04821.1/ AAG04819.1 |
| RhlI/R | *P. aeruginosa* PAO1 | C4-HSL | AAG06864.1/ AAG06865.1 |
| PfvI/R | *Pseudomonas fuscovaginae* UPB0736 | 3OC10/ 3OC12-HSL | CAQ15950.2/ CAQ15948.2 |
| PfsI/R | *P. fuscovaginae* UPB0736 | C10/ C12-HSL | CBI67625.1/ CBI67623.1 |
| CepI/R | *Burkholderia vietnamiensis* G4 | C8-HSL | ABO58211.1/ ABO58209.1 |
| BviI/R | *B. vietnamiensis* G4 | C10-HSL | ABK32009.1/ ABK32010.1 |
| BtaI/R1 | *Burkholderia thailandensis* E264 | C8-HSL | ABC35524.1/ ABC34804.1 |
| BtaI/R2 | *B. thailandensis* E264 | 3OHC10-HSL | ABC34067.1/ ABC34774.1 |
| BtaI/R3 | *B. thailandensis* E264 | 3OHC8-HSL | AIP27810.1/ AIP27980.1 |
| CepI/R | *Burkholderia cenocepacia* J2315 | C8-HSL | CAR55728.1/ CAR55726.1 |
| AbaI/R | *Acinetobacter baumannii* ATCC17978 | 3OHC12-HSL | AKQ28471.1/ AKQ28469.1 |
| CviI/R | *Chromobacterium violaceum* ATCC 12472 | 3OHC10-HSL | AAQ61751.2/ AAQ61750.2 |
| CviI/R | *Chromobacterium violaceum* ATCC 31532 | C6-HSL | PLV42917.1/ ADC79709.1 |
| LuxI/R | *Vibrio fischeri* ES114 | 3OC6-HSL | AAW87994.1/ AAW87995.1 |
| LuxI/R^a^ | *Vibrio fischeri* MJ11 | 3OC6-HSL | ACH64323.1/ ACH63788.1 |
| ExpI/R | *Pectobacterium parmentieri* SCC3193 | 3OC8-HSL | AFI92653.1/ AFI92652.1 |
| BjaI/R | *Bradyrhizobium japonicum* USDA110 | Isovaleryl-HSL | BAC46328.1/ BAC46327.1 |
| MupI/R | *Pseudomonas fluorescens* NCIMB 10586 | 3OC10-HSL | AAK28505.1/ AAK28504.1 |
| PpuI/R | *Pseudomonas putida* IsoF | 3OC10/3OC12-HSL | AAM75411.1/ AAM75413.1 |
| TraI/R | *Agrobacterium tumefaciens* C-58 | 3OC8-HSL | AAK91000.1/ AAK91098.1 |
| MbaI/R | *Methylobacter tundripaludum* 21/22 | 3OHC10-HSL | WP_150113271.1/ WP_006890625.1^b^ |
| YruI/R | *Yersinia ruckeri* ATCC 29473 | 3OC8-HSL | KGA49182.1/ KGA49159.1 |
| GtaI/R | *Rhodobacter capsulatus* SB1003 | C16-HSL | ADE84094.1/ ADE84093.1 |
| AhyI/R | *Aeromonas hydrophila* ML09-119 | C4-HSL | AGM42354.1/ AGM42355.1 |

^a^ Covariation analyses were mapped onto LasI/R from *P. aeruginosa* PAO1 and LuxI/R from *V. fischeri* MJ11.

^b^ The accession number provided is for the NCBI Reference Sequence.

**Supplementary File 1B | Select previously reported data for LasR homologs with relevant amino acid substitutions**. The native signal for each receptor is indicated in parentheses.

| LasR residue | LasR  homolog | Mutation | Effect on receptor activity**^a^** | Reference |
| --- | --- | --- | --- | --- |
| **L36** | LuxR  (3OC6-HSL) | L42A^b^ | Reduced sensitivity | (Koch et al., 2005) |
|  |  | L42S | Reduced sensitivity |  |
|  | TraR^c^  (3OC8-HSL) | A38V | Reduced activity | (Luo et al., 2003) |
| **G38** | QscR (3OC12-HSL) | G40F | Altered selectivity: impaired response to 3OC12-HSL, increased sensitivity to 3OC6-HSL | (Lintz et al., 2011) |
| **P57** | TraR | H54Y | Reduced activity | (Luo et al., 2003) |
| **R61** | LasR (3OC12-HSL) | R61M | Altered selectivity: impaired response to 3OC12-HSL, maintained response to C12-HSL | (Collins et al., 2006) |
|  | LasR | R61M | Reduced sensitivity | (Gerdt et al., 2015) |
|  | LuxR | R67M | Altered selectivity: impaired response to 3OC6-HSL, maintained response to C6-HSL | (Collins et al., 2006) |
|  | TraR^c^ | Q58L | Altered selectivity: improved sensitivity to 3OC6-HSL | (Chai and Winans, 2004) |
|  |  | Q58F | Reduced activity |  |
| **T75** | LasR | T75V | Increased sensitivity | (Gerdt et al., 2015) |
| **V76** | QscR | V78F | Reduced activity | (Lintz et al., 2011) |
| **A127** | LasR | A127W | Altered selectivity: impaired response to 3OC12-HSL, maintained response to shorter AHLs | (McCready et al., 2019b) |
|  | LasR | A127F | Reduced sensitivity | (McCready et al., 2019a) |
|  | LuxR | M135I | Altered selectivity: impaired response to 3OC6-HSL; improved response to C8-HSL | (Collins et al., 2005) |
|  |  | M135V |  |  |
|  | LuxR | M135A | Reduced sensitivity | (Koch et al., 2005) |
| **S129** | LasR | S129A | Reduced sensitivity | (Gerdt et al., 2015; Manson et al., 2020) |
|  | LasR | S129C | Reduced sensitivity | (McCready et al., 2019b) |
|  |  | S129W | Reduced sensitivity |  |
|  |  | S129F | Reduced sensitivity |  |
|  |  | S129T | Reduced sensitivity |  |
|  |  | S129M | Reduced sensitivity |  |
|  | TraR | T129S | No change from wild-type | (Chai and Winans, 2004) |
|  |  | T129L | Reduced activity |  |
|  |  | T129I | Reduced activity |  |
|  |  | T129F | Reduced activity |  |
|  |  | T129A | Altered selectivity: impaired activity; equally sensitive to C8-HSL and 3OC8-HSL |  |
|  |  | T129V |  |  |
| **L130** | LasR | L130F | Altered selectivity: increased sensitivity to 3OC12-HSL and to other AHLs | (McCready et al., 2019b) |

^a^ All studies measured the activity of the receptor in *Escherichia coli* unless otherwise noted.

^b^ Numbering is according to the *Vibrio fischeri* ES114 sequence.

^c^ This study was performed in *Agrobacterium tumefaciens*, using a *traR* expression plasmid.

**Supplementary File 1C | Select previously reported data for LasI homologs with relevant amino acid substitutions.** The native signal for each synthase is indicated in parentheses.

| LasI residue | LasI homolog | Mutation | Effect on synthase activity**a** | Reference |
| --- | --- | --- | --- | --- |
| **S103** | RhlI  (C4-HSL) | S103E | Impaired activity | (Parsek et al., 1997) |
|  | EsaI  (3OC6-HSL) | S99A | Impaired activity | (Watson et al., 2002) |
| **T142** | LasI  (3OC12-HSL) | T142G | Impaired activity | (Gould et al., 2006) |
|  |  | T142A | Slightly altered selectivity |  |
|  |  | T142S | Slightly altered selectivity |  |
|  | EsaI  (3OC6-HSL) | T140A | Altered selectivity: Increased production of C6-HSL | (Gould et al., 2006; Watson et al., 2002) |
|  | EsaI | T140V | Impaired activity | (Watson et al., 2002) |
| **T144** | LasI | T144V | Impaired activity | (Gould et al., 2006) |
| **M152** | RhlI | F147Lb | Increased activity | (Kambam et al., 2009) |
|  | MesIc  (C6-HSL) | L153A | Altered selectivity: Increased C8-HSL production | (Dong et al., 2020) |
|  |  | L153F | Altered selectivity: Increased C4-HSL production |  |
|  | BjaI^c^  (isovaleryl-HSL) | F147Y | Altered selectivity: Increased C4-HSL production | (Dong et al., 2020) |

a All studies measured activity of the synthase in *Escherichia coli* unless otherwise noted.

b F147L was one of three amino acid substitutions in a synthase obtained by directed evolution.

c Substrate selectivity was studied using purified enzymes.

**Supplementary File 1D | LasR amino acid substitutions evaluated in Figure 3.** Relative abundance is the frequency of a given amino acid at the indicated position across all LasR homologs.

| **Residue in receptor** | **Amino acid** | **Relative abundance** |
| --- | --- | --- |
| 38 | Gly* | 0.482 |
|  | Leu | 0.095 |
|  | Val | 0.086 |
|  | Ala | 0.079 |
| 61 | Val | 0.231 |
|  | Gln | 0.160 |
|  | Leu | 0.128 |
|  | Met | 0.126 |
|  | Arg* | 0.093 |
| 127 | Leu | 0.287 |
|  | Met | 0.174 |
|  | Ala* | 0.102 |
| 129 | Ser* | 0.536 |
|  | Thr | 0.273 |
|  | Ala | 0.092 |
|  | Asn | 0.049 |
| 130 | Leu* | 0.359 |
|  | Phe | 0.253 |
|  | Ile | 0.176 |

* Indicates identity in wild-type LasR.

**Supplementary File 1E | LasI amino acid substitutions evaluated in Figure 5 – figure supplement 1.** Relative abundance is the frequency of a given amino acid at the indicated position across all LasI homologs.

| **Residue in synthase** | **Amino acid** | **Relative abundance** |
| --- | --- | --- |
| 102 | Leu* | 0.398 |
|  | Ser | 0.157 |
|  | Met | 0.156 |
|  | Ile | 0.094 |
| 142 | Gly | 0.379 |
|  | Thr* | 0.314 |
|  | Ala | 0.154 |
| 145 | Ser | 0.231 |
|  | Thr* | 0.199 |
|  | Pro | 0.170 |
|  | Asp | 0.135 |
|  | Ala | 0.009 |
| 157 | Trp | 0.271 |
|  | Val | 0.220 |
|  | Leu* | 0.124 |

* Indicates identity in wild-type LasI.

**Supplementary File 1F | Primers used in this study.**

| Primer Name | Sequence | Description |
| --- | --- | --- |
| lasI-pJN-F | TTGGGCTAGCATGATCGTACAAATTGG | Amplifies *lasI* adding homology to pJN105. *lasI* sequence underlined |
| lasI-pJN-R | TTGGAGCTCCTCATGAAACCGCCAGTC | Amplifies *lasI* adding homology to pJN105, including SacI site. *lasI* sequence underlined. |
| RBS-lasI-F | TTGGGCTAGCAAGGAGGAAGTGAAGATGATCGTACAAATTG | Amplifies *lasI*, including RBS, adding NheI site. *lasI* sequence underlined. |
| mupI-F | TTGGGCTAGCAAGGAGGAAGCCAGCATGAAATATCTAATAG | Amplifies *mupI*, adding RBS and NheI site. *mupI* sequence underlined. |
| mupI-R | TTGGAGCTCTCAAATAGCATTGACTGCGTCC | Amplifies *mupI*, adding SacI site. *mupI* sequence underlined. |
| mupI-pJN-F | TCCATACCCGTTTTTTTGGGCTAGCAAGGAGGAAGCCAG | Amplifies *mupI* PCR product produced by mupI-F & mupI-R, adding additional homology to pJN105 |
| mupI-pJN-R | CTCACTATAGGGCGAATTGGAGCTCCTCAAATAGCATTG | Amplifies *mupI* PCR product produced by mupI-F & mupI-R, adding additional homology to pJN105. *mupI* sequence underlined. |
| mupR-F | GCAAGGAGGAAGCCAGCATGCTTGAAGACATTCTGATG | Amplifies *mupR*, adding homology to pJN105. *mupR* sequence underlined. |
| mupR-R | GCGAATTGGAGCTCCTCAGGGCGTGACC | Amplifies *mupR*, adding homology to pJN105. *mupR* sequence underlined. |
| P_mupI_-F | GTACAAGCGCAGCAGCATGTAC | Amplifies *mupI* promoter from -300 relative to the translational start site |
| P_mupI_-R | ACGTGGTGATATGTTCTCGCGTTTG | Amplifies *mupI* promoter from +42 relative to the translational start site |
| P_mupI_-pPR-F | GCATGCCTGCAGGTCGACGTACAAGCGCAGCAGCATG | Amplifies *mupI* promoter, adding homology to pPROBE-GT. *mupI* promoter sequence underlined. |
| P_mupI_-pPR-R | CTCGGTACCCGGGGATCCACGTGGTGATATGTTCTCGC | Amplifies *mupI* promoter, adding homology to pPROBE-GT. *mupI* promoter sequence underlined. |

*Site-directed mutagenesis primer sequences are available upon request.*

**References**

Chai Y, Winans SC (2004) **Site-directed mutagenesis of a LuxR-type quorum-sensing transcription factor: Alteration of autoinducer specificity**. *Molecular Microbiology* **51**: 765-776.

Collins CH, Arnold FH, Leadbetter JR (2005) **Directed evolution of *Vibrio fischeri* LuxR for increased sensitivity to a broad spectrum of acyl-homoserine lactones**. *Molecular Microbiology* **55**: 712-723.

Collins CH, Leadbetter JR, Arnold FH (2006) **Dual selection enhances the signaling specificity of a variant of the quorum-sensing transcriptional activator LuxR**. *Nature Biotechnology* **24**: 708-712.

Dong S-H, Nhu-Lam M, Nagarajan R, Nair SK (2020) **Structure-guided biochemical analysis of quorum signal synthase specificities**. *ACS Chemical Biology* **15**: 1497-1504.

Gerdt JP, McInnis CE, Schell TL, Blackwell HE (2015) **Unraveling the contributions of hydrogen-bonding interactions to the activity of native and non-native ligands in the quorum-sensing receptor LasR**. *Organic & Biomolecular Chemistry* **13**: 1453-1462.

Gould TA, Herman J, Krank J, Murphy RC, Churchill MEA (2006) **Specificity of acyl-homoserine lactone synthases examined by mass spectrometry**. *Journal of Bacteriology* **188**: 773-783.

Kambam PKR, Eriksen DT, Lajoie J, Sayut DJ, Sun L (2009) **Altering the substrate specificity of RhlI by directed evolution**. *ChemBioChem* **10**: 553-558.

Koch B, Liljefors T, Persson T, Nielsen J, Kjelleberg S, Givskov M (2005) **The LuxR receptor: The sites of interaction with quorum-sensing signals and inhibitors**. *Microbiology* **151**: 3589-3602.

Lintz MJ, Oinuma K-I, Wysoczynski CL, Greenberg EP, Churchill MEA (2011) **Crystal structure of QscR, a *Pseudomonas aeruginosa* quorum sensing signal receptor**. *Proceedings of the National Academy of Sciences* **108**: 15763-15769.

Luo Z-Q, Smyth AJ, Gao P, Qin Y, Farrand SK (2003) **Mutational analysis of TraR: Correlating function with molecular structure of a quorum-sensing transcriptional activator**. *Journal of Biological Chemistry* **278**: 13173-13182.

Manson DE, O’Reilly MC, Nyffeler KE, Blackwell HE (2020) **Design, synthesis, and biochemical characterization of non-native antagonists of the *Pseudomonas aeruginosa* quorum sensing receptor LasR with nanomolar IC50 values**. *ACS Infectious Diseases* **6**: 649-661.

McCready AR, Paczkowski JE, Cong J-P, Bassler BL (2019a) **An autoinducer-independent RhlR quorum-sensing receptor enables analysis of RhlR regulation**. *PLoS Pathogens* **15**: e1007820.

McCready AR, Paczkowski JE, Henke BR, Bassler BL (2019b) **Structural determinants driving homoserine lactone ligand selection in the *Pseudomonas aeruginosa* LasR quorum-sensing receptor**. *Proceedings of the National Academy of Sciences* **116**: 245-254.

Parsek MR, Schaefer AL, Greenberg EP (1997) **Analysis of random and site-directed mutations in *rhlI*, a *Pseudomonas aeruginosa* gene encoding an acylhomoserine lactone synthase**. *Molecular Microbiology* **26**: 301-310.

Watson WT, Minogue TD, Val DL, von Bodman SB, Churchill MEA (2002) **Structural basis and specificity of acyl-homoserine lactone signal production in bacterial quorum sensing**. *Molecular Cell* **9**: 685-694.
